# Reduced nicotine content cigarettes in smokers of low socioeconomic status: study protocol for a randomized control trial

**DOI:** 10.1186/s13063-017-2038-9

**Published:** 2017-07-03

**Authors:** Nicolle M. Krebs, Sophia I. Allen, Susan Veldheer, Diane J. Martinez, Kimberly Horn, Craig Livelsberger, Jennifer Modesto, Robin Kuprewicz, Ashley Wilhelm, Shari Hrabovsky, Abid Kazi, Alyse Fazzi, Jason Liao, Junjia Zhu, Emily Wasserman, Samantha M. Reilly, Lisa Reinhart, Neil Trushin, Robinn E. Moyer, Rebecca Bascom, Jonathan Foulds, John P. Richie, Joshua E. Muscat

**Affiliations:** 1 Department of Public Health Sciences, Penn State Tobacco Center of Regulatory Science, Pennsylvania State University, MC CH69, 500 University Drive, P.O. Box 850, Hershey, PA 17033 USA; 2 Investigational Drug Service, Department of Pharmacy, Pennsylvania State University, 500 University Drive, P.O. Box 850, Hershey, PA 17033 USA; 3 The Milken School of Public Health, George Washington University, 950 New Hampshire Ave. NW, Washington, D.C, 20052 USA; 4 Department of Medicine, Pennsylvania State University, 500 University Drive, P.O. Box 850, Hershey, PA 17033 USA

**Keywords:** Tobacco, Cigarettes, Smoking, Randomized controlled trial, Cotinine, Reduced nicotine content cigarettes, Socioeconomic status, Education, Tobacco control

## Abstract

**Background:**

The Family Smoking Prevention and Tobacco Control Act gave the Food and Drug Administration jurisdiction over the regulation of all tobacco products, including their nicotine content. Under this act, a major strategy to reduce harm from cigarette tobacco is lowering the nicotine content without causing unintended adverse consequences. Initial research on reduced nicotine content (RNC) cigarettes has shown that smokers of these cigarettes gradually decrease their smoking frequency and biomarkers of exposure. The effectiveness of this strategy needs to be demonstrated in different populations whose response to RNC cigarettes might be substantially mediated by personal or environmental factors, such as low socioeconomic status (SES) populations. This study aims to evaluate the response to a reduced nicotine intervention in low SES smokers, as defined here as those with less than 16 years of education, by switching smokers from high nicotine commercial cigarettes to RNC cigarettes.

**Methods/design:**

Adults (*N* = 280) who have smoked five cigarettes or more per day for the past year, have not made a quit attempt in the prior month, are not planning to quit, and have less than 16 years of education are recruited into a two-arm, double-blinded randomized controlled trial. First, participants smoke their usual brand of cigarettes for 1 week and SPECTRUM research cigarettes containing a usual amount of nicotine for 2 weeks. During the experimental phase, participants are randomized to continue smoking SPECTRUM research cigarettes that contain either (1) usual nicotine content (UNC) (11.6 mg/cigarette) or (2) RNC (11.6 to 0.2 mg/cigarette) over 18 weeks. During the final phase of the study, all participants are offered the choice to quit smoking with nicotine replacement therapy, continue smoking the research cigarettes, or return to their usual brand of cigarettes. The primary outcomes of the study include retention rates and compliance with using only research cigarettes and no use of other nicotine-containing products. Secondary outcomes are tobacco smoke biomarkers, nicotine dependence measures, smoking topography, stress levels, and adverse health consequences.

**Discussion:**

Results from this study will provide information on whether low SES smokers can maintain a course of progressive nicotine reduction without increases in incidence of adverse effects.

**Trial registration:**

ClinicalTrials.gov, NCT01928719. Registered on 21 August 2013.

**Electronic supplementary material:**

The online version of this article (doi:10.1186/s13063-017-2038-9) contains supplementary material, which is available to authorized users.

## Background

Reducing the addictiveness of cigarettes by lowering the nicotine content is a proposed national regulatory policy [1] that is permissible under the Family Smoking Prevention and Tobacco Control Act (TCA) of 2009 [2]. This act gave the Food and Drug Administration (FDA) the authority to reduce, but not completely eliminate, nicotine in tobacco products. This policy could only be enacted if it benefits public health by lowering harm due to tobacco exposure. If the levels of nicotine in cigarettes were reduced to levels that were not addictive, young individuals who have an interest in smoking might never develop a dependence on cigarettes, the leading cause of morbidity and premature mortality in the USA [3]. The Institute of Medicine and the US Office of the Surgeon General indicate that a nicotine reduction strategy is feasible and guidelines need to be developed to implement this policy [4, 5]. However, potential harms from this strategy could include (1) the physiological and psychological conditions associated with nicotine withdrawal and (2) the possibility that smokers may increase their toxicant inhalation by smoking more in order to compensate for the reduced nicotine per cigarette (compensatory smoking).

Cigarette smoking is predominantly a health concern among persons with lower income and less education [6], referred to herein as persons with a low socioeconomic status (SES). When considering a national policy, it is necessary to consider one of the greatest predictors of tobacco use, which is low SES [7]. More than one quarter of adults below the poverty level smoke tobacco, compared to only 14% at or above the poverty level [8]. Cigarette smoking is the highest among adults with a graduate education degree (GED) certificate (34%) and lowest among college graduates (3–7%) [8]. Low SES populations not only have high levels of smoking [7] but also high levels of other unhealthy behaviors, such as poor eating choices and lower levels of physical activity [9–12]. This may present unique challenges to a nicotine reduction strategy. Disparities in smoking cessation outcomes include smokers with less education are less likely to intend to quit, initiate a quit attempt, or be abstinent from smoking for at least 1 month [13]. The lack of intention to quit and the inability to quit among low SES smokers contributes to the clear gradient in smoking prevalence by income and education. The differences in smoking rates between low and high SES populations are projected to deviate even more in the future [14]. Barriers to quitting among low SES smokers include psychosocial factors (attitude, social norm, self-efficacy) [15–17], higher stress levels [18], less social support [19, 20], and an inaccessibility to treatment [21, 22].

Smoking is a behavior that is reported to relieve stress [23–26]. However, the stress hormone, cortisol, is shown to be elevated in smokers compared to non-smokers [27], and perceived stress levels reduce after smokers quit [28]. The elevation of cortisol in smokers is attributed to nicotine exposure [29, 30]. As an additive influence, cortisol also has been shown to be elevated in low SES populations, although the findings are not consistent [31]. Elevated levels of stress hormones can have a deleterious effect on the body [32]. An assessment of whether the gradual reduction in nicotine changes the stress hormones in the body is another focus of this study.

Research evaluating reduced nicotine in cigarettes has been published [33–39]. In an initial study of switching smokers to research cigarettes with very low nicotine content [34], participants were asked to smoke their usual brand of cigarettes and one of five research cigarettes with varying nicotine contents in a progressive manner (range: 12 to 1 mg/cigarette). At the end of the study, participants reduced their cigarettes per day, nicotine dependence, cravings, and biomarkers of nicotine [34]. In a 6-month controlled trial of progressively lower nicotine content cigarettes, 135 current smokers [36] demonstrated reduced exposure to tobacco constituents (including nicotine) without increases in cigarette consumption or tobacco smoke byproducts (carbon monoxide and polycyclic aromatic hydrocarbons). However, the mean level of education among the participants was college graduate (e.g., ~15–16 years of education). An alternative strategy from progressively switching from high to low nicotine cigarettes is a non-progressive switch to low nicotine cigarettes. In a 6-week study examining the non-progressive approach, reduced nicotine cigarette use resulted in reduced nicotine exposure, reduced nicotine dependence, and reduced biomarkers [37]. However, compliance in only using research cigarettes in these trials is a concern [40], and although the extent of non-compliance was small for many participants, the feasibility of a nicotine reduction strategy depends on balancing the need for reducing dependence and the incidence of tobacco-related disease and minimizing the potential harms to smokers associated with withdrawal. In a national nicotine reduction policy, smokers would not have available their full-strength nicotine cigarettes, and potential harms from this policy include the loss of the psychological benefits of smoking, a dislike of the taste of low-nicotine cigarettes, and potential short-term compensation [35, 39, 41–45].

The premise of nicotine reduction is to lower the nicotine content in cigarettes to non-addicting levels, which could in theory reduce tobacco addiction in current smokers and eliminate the onset of dependent cigarette smoking in young people. Commercial cigarettes typically contain about 8–9 mg of nicotine per cigarette [46], and the delivery of nicotine per cigarette to the smoker is around 1 mg, but it can vary [47]. Based on the bioavailability of nicotine, a reduced nicotine content (RNC) of 0.5 mg per cigarette has been proposed as an addictive cut point [1] and may make it easier for established smokers to quit [38]. This differs from smokers switching to “light” cigarettes, a label no longer allowed under the TCA. Light cigarettes reduce machine-smoked nicotine and tar yields by various design changes that increase the ventilation or air flow through the cigarette such as adding perforations in the filter. The light cigarettes had similar nicotine content to regular cigarettes, and smokers could cover the ventilation holes to increase their nicotine delivery and circumvent the design feature [48–51]. In contrast, RNC cigarettes actually contain less nicotine, and compensation is not as feasible, especially at very low levels of nicotine content. However, some compensation may still occur if smokers compensate by smoking more of an individual cigarette or more cigarettes per day. If high nicotine cigarettes were no longer available in the marketplace, smokers might compensate by purchasing and consuming high nicotine black market cigarettes [52]. It could be expected that, without the ability to compensate, smokers would find low nicotine cigarettes unacceptable. However, trials of switching to reduced nicotine research cigarettes have been shown to be successful, although biomarker data suggest some level of non-compliance as determined by the ratio of cotinine, the immediate metabolite of nicotine, to the number of research cigarettes smoked [53]. Significant predictors of non-compliance were cigarette satisfaction, dependence, and age. More data are needed to assess the feasibility of this regulatory policy in high-risk smokers, especially smokers who have a high degree of dependence, such as low SES populations. The study described here aims to test the capability of a nicotine reduction intervention, where low SES smokers are switched from their usual brand of commercial cigarettes to progressively lower nicotine content cigarettes in a randomized controlled trial.

## Methods/design

### Study population

Cigarette smokers with less than 16 years of education, with access to the Penn State Milton S. Hershey Medical Center Hershey, in Hershey, PA or the George Washington University Milken Institute School of Public Health, in Washington, DC, who report no quit attempt in the past month and do not plan to quit smoking in the next 6 months are eligible. Additional eligibility criteria are outlined in Table 1.

**Table 1:** Inclusion and exclusion criteria

Inclusions:	
Age	18–65
Education	<16 years or < Bachelor’s degree
Cigarette frequency	≥5 cigarettes per day
Cigarette history	Continuously for at least the last 12 months
Cigarette flavor	Willing to smoke one flavor of cigarette (menthol or non-menthol)
Comprehension	Able to read and write in English and to understand and consent to study procedures
Smoking cessation	Made no serious quit attempt with/without pharmacotherapy in the prior 1 month and have no plans to quit in the next 6 months
Availability	Accessibility to study centers and to receive phone calls for the next 8 months
Exclusions:	Current pregnancy or nursing, unstable or serious medical conditions, prisoners or subject to correctional supervision, systolic blood pressure ≥160 mmHg, use of non-cigarette nicotine delivery product in the past week, difficulty providing blood samples, regular use of illegal drugs, inpatient treatment for substance abuse or mental health condition in past 6 months, alcohol abuse hindering person’s ability to participate, other members of the household currently participating in a trial related to reduced nicotine cigarettes, major surgery planned in the next 8 months (not including outpatient), or any other factor that may affect adherence or pose a health risk to the participant

### Design

**Fig. 1:**
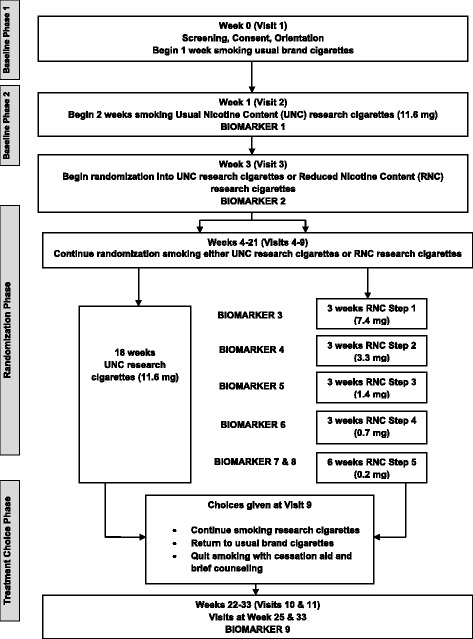
Study flow diagram

This is a two-arm, double-blind, parallel-group, randomized controlled trial. The study consists of four phases (Baseline I, Baseline II, Randomization, and Treatment Choice) over 34 weeks (Fig. 1). Participants are randomized into either a Usual Nicotine Content (UNC) cigarette or a Reduced Nicotine Content (RNC) cigarette treatment group. Table 2 shows the nicotine content dosing schedule for the two treatment groups. Participants and study staff are blind to the cigarette treatment group throughout the trial. Visits occur during the day at the Penn State University College of Medicine Clinical Research Center in Hershey, PA and George Washington University Milken School of Public Health and Medical Faculty Associates in Washington, DC.

**Table 2:** Nicotine content dosing schedule

Phase	Baseline Phase I	Baseline Phase II	Randomized Double-Blind Phase					Treatment Choice Phase
Week(s)	1	2	3	3	3	3	6	12
RNC cigarette group	Own brand	Usual nicotine research cigarettes	Reduced nicotine step 1	Reduced nicotine step 2	Reduced nicotine step 3	Reduced nicotine step 4	Reduced nicotine step 5	Variable
UNC cigarette group	Own brand	Usual nicotine research cigarettes	Usual nicotine research cigarettes	Usual nicotine research cigarettes	Usual nicotine research cigarettes	Usual nicotine research cigarettes	Usual nicotine research cigarettes	Variable
*Approximate nicotine content in mg/cigarette (mg/gram)* ^a^								
RNC cigarette group	13^b^ (19)	11.6 (16.5)	7.4 (10.6)	3.3 (4.7)	1.4 (1.9)	0.7 (0.9)	0.2 (0.3)	Variable
UNC cigarette group	13^b^ (19)	11.6 (16.5)	11.6 (16.5)	11.6 (16.5)	11.6 (16.5)	11.6 (16.5)	11.6 (16.5)	Variable

*Abbreviations*: *RNC* reduced nicotine content, *UNC* usual nicotine content

SPECTRUM Tobacco Product Master File Codes used for RNC group (non-menthol/menthol) are NRC600/NRC601 or NRC602, NRC500/NRC501, NRC400/NRC401, NRC300/NRC301, NRC200/NRC201, and NRC102/NRC103. SPECTRUM Tobacco Product Master File Codes used for UNC group (non-menthol/menthol) are NRC600/NRC601 or NRC602

^a^These are averages of menthol/non-menthol cigarettes at each level based on estimated 0.7 g tobacco content per cigarette and nicotine concentrations based on Richter et al. (2016) [54]

^b^Approximate mean nicotine content and concentration of commercially available cigarettes based on Connolly et al. (2007) [62]

#### Primary Aim

The primary aim of the trial is to determine adherence to a regimen of progressively lowering cigarette nicotine levels. Efficacy will be determined by the ability to complete the study and degree of compliance in the UNC versus RNC treatment groups. The participant’s compliance with using only the research cigarettes and no other nicotine-containing products including their usual brand of cigarettes will be determined by both self-report and biochemical measures. We hypothesize that non-compliance and drop-out rates will be higher in the RNC versus the UNC treatment group.

#### Secondary Aims

The secondary aims are as follows:

1. Determine the effect of progressive nicotine content reduction on nicotine metabolites and other biomarkers of smoking exposure. We hypothesize that the gradual reduction of nicotine from progressively lowering nicotine exposure will lead to lower levels of blood nicotine metabolites and nicotine-derived carcinogens, while not affecting overall cigarettes smoked per day
  Determine the modifying effect of menthol on progressive nicotine content reduction and biomarkers. We hypothesize that menthol will not modify the effect of progressive RNC cigarettes on the above biomarkers
2. Determine if a gradual reduction in nicotine content in RNC cigarettes is associated with a reduction in stress. We hypothesize that the reduction in nicotine will lead to a reduction in levels of psychological stress and stress biomarkers
3. Determine if smoking topography measures change in response to RNC versus UNC cigarettes. We hypothesize that smoking topography measures (i.e., puff volume, puff count, puff flow, and puff duration) will increase for smokers in the RNC cigarette group due to compensatory smoking

##### Primary outcomes

The primary outcomes are the following:

- Non-compliance (use of other nicotine-containing products) by biochemical and self-report measures during the randomization phase
- Participant drop-out rate during the randomization phase

##### Secondary outcomes

Secondary outcomes (measured from baseline to the end of the randomization phase) are:

- Changes in cigarettes per day
- Changes in nicotine (measured by plasma cotinine) and tobacco smoke exposure (measured by expired carbon monoxide, tobacco-specific *N*-nitrosamines) biomarkers
- Changes in measures of oxidative stress including glutathione (oxidized:reduced ratio) and 8-isoprostanes
- Changes in nicotine dependence and withdrawal symptoms
- Changes in stress (in subgroup), measured by the Perceived Stress Questionnaire and salivary cortisol and alpha amylase
- Changes in smoking topography measures (in subgroup), measured by puff volume, puff count, puff flow, puff duration, inter-puff interval
- Changes in adverse health effects (e.g., high blood pressure, adverse respiratory symptoms)
- Proportion of participants choosing to make a quit attempt with biochemically verified abstinence

### Research cigarettes

SPECTRUM research cigarettes are available only through the National Institute of Drug Abuse Drug Supply Program (NOT-DA-14-004) and are obtained via an Investigational Tobacco Product (ITP) application. The physical properties of these cigarettes are described elsewhere [54]. An ITP is defined as “a new or modified risk tobacco product that is not legally marketed; or a tobacco product that is required to comply with a tobacco product standard and that does not conform in all respects to the applicable tobacco product standard and is intended for investigational use.” An ITP application requires a detailed protocol submission and plans for adherence to the product usage. SPECTRUM research cigarettes in regular and menthol flavors are shipped from the Research Triangle Institute, Research Triangle Park, NC, USA. Cartons contain 10 packs of cigarettes, and each pack contains 20 cigarettes. Each cigarette is approximately 83 mm long. All cigarette packs are labeled with a Surgeon General’s Warning and a “for research purposes only” indication.

### Blind coding of the research cigarettes

SPECTRUM cigarette cartons are shipped to the Department of Pharmacy at Penn State Milton S. Hershey Medical Center. Each carton received comes with the manufacturer’s barcoded labels that identify the nicotine content and a batch/lot number. Individual packs and cigarettes do not contain any identifiable labels. The manufacturer’s labels are recorded and removed from the carton and replaced with a blind code number by an unblinded cigarette administrator who has no direct contact with participants. Each carton is packaged with a sheet of 10 additional labels of the same blind code number to be affixed to the individual packs within the carton. The link between the blind code number and the nicotine content of each carton is housed in our proprietary Cigarette Management System (CMS), which allows the cigarette administrator to dispense appropriate cartons to participants in a blinded fashion. The computer-generated randomization sequence is housed within the CMS and stratified by study site and cigarette flavor (regular or menthol). Within each of the strata a block randomization is used. Unblinding of the cigarette allocation is only permissible if it becomes medically necessary for the safety of the participant.

### Early withdrawal of participants

This study is designed to identify participants who are not able or unwilling to comply with the full study protocol by having two baseline phases (I and II) prior to randomization. During Baseline Phase I, participants smoke their usual brand of cigarettes for 1 week. At Baseline Phase II, all participants smoke SPECTRUM research cigarettes with normal nicotine content (about 11.6 mg).

During Baseline Phases I and II, participants are withdrawn if they report smoking other products or using other nicotine-containing products at more than one visit. This includes any number of usual brand cigarettes (when research cigarettes are received), cigars, pipes, snuff, chew, hookahs, electronic cigarettes, marijuana, or any other illegal smoked substance or nicotine-containing product.

During Baseline Phase II, participants are withdrawn if:

1. The total cigarette consumption includes more than 10% of non-research cigarettes.
2. The participant has reduced his/her cigarette consumption by more than 50% compared to baseline cigarettes per day. This does not include situations such as illness.

Participants who are removed prior to randomization (during Baseline Phases I and II) are replaced until a total of 280 participants have been randomized.

Participants are monitored and may be withdrawn if any of the following occurs:

1. Expired breath carbon monoxide (CO) increases from baseline according to the following:
  - CO level is greater than 50 ppm if CO at baseline is <20 ppm.
  - CO level is greater than 60 ppm if CO at baseline is 20–34 ppm.
  - CO level is greater than 70 ppm if CO at baseline is 35–49 ppm.
  - CO level is greater than 80 ppm if CO at baseline is 50–60 ppm.
  - CO level is greater than 90 ppm if CO at baseline is 61–70 ppm.
2. Cigarettes per day increases by more than 100% from average cigarettes per day at baseline
3. Increase in systolic blood pressure above 160 mmHg
4. Increase in substance abuse
5. Any hospitalization or debilitation in which participation in the study could be detrimental to the recovery process
6. Any missed visits where research cigarettes are given
7. Any situation where the participant is not able to smoke the research cigarettes for a period of more than 2 weeks
8. Participant behavior demonstrates an inability to continue with the study

Participant withdrawal will occur at any point during the study if the participant becomes pregnant, the participant suffers from a serious medical condition (e.g., heart attack, stroke, blood clots), the participant decides to withdraw, or for any other condition or situation that would, in the investigator’s opinion, make it unlikely that the participant could comply with the study protocol.

### Recruitment process

Participants are recruited throughout the Hershey, PA and Washington, DC areas by using traditional recruitment methods (i.e., mailed and posted flyers, printed and radio advertisements, community outreach events) in addition to Internet and social media platforms (Craigslist, Facebook) and word of mouth. They are screened for eligibility over the phone and invited to the study centers where they are further assessed for eligibility.

### Procedures

**Fig. 2:**
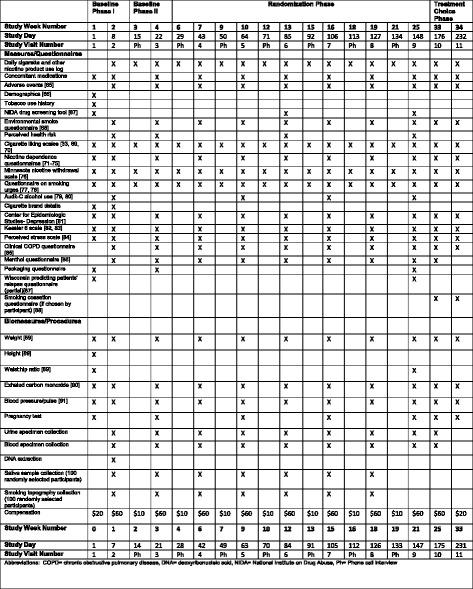
SPIRIT diagram

The study flow diagram is given in Fig. 1. See Additional file 1 for the Standard Protocol Items: Recommendations for Interventional Trials (SPIRIT) checklist. Participants complete biomeasures, questionnaires, and procedures as outlined in Fig. 2 (the SPIRIT diagram). The Penn State Tobacco Center of Regulatory Science (TCORS) Biomarker Core will perform the analysis on all biological samples (blood, urine, saliva). Study visit procedures are described in the following subsections.

#### Baseline Phase I: 1 week

During Visit 1, final eligibility determination and informed consent are obtained from the participant by trained study staff, and the usual discussion of procedures, risks, side effects, confidentiality, voluntary participation, and right to refuse participation without prejudice is explained to the participant. Participants must be capable of understanding the nature of this study and its potential risks, discomforts, and benefits before signing the consent form. After consent is obtained, the study staff screens for drug abuse, obtains medical and concomitant medication histories, and measures vital signs, with eligibility determination based on the inclusion/exclusion criteria listed in Table 1. For women of childbearing age, a urine sample is collected for a pregnancy test.

**Fig. 3:**
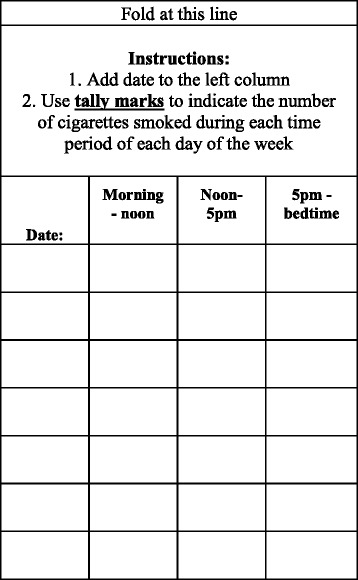
Participants are instructed to keep their daily cigarette log attached to their cigarette pack by folding the top of the log at the line and tucking the flap into the pack

Once a participant is determined eligible, further biomeasures are obtained (i.e., exhaled CO). Participants are asked to complete Visit 1 questionnaires (Fig. 2) on a computer, and the study staff reviews the study guidelines and provides participants with instructions on how to keep track of the number of usual brand cigarettes smoked each day by using a cigarette log (Fig 3). A randomly selected subset of 200 participants is asked to complete either a saliva sample collection (50 at each study site) or a smoking topography protocol (50 at each study site). If a participant is chosen to complete the saliva sample collection, he/she is provided with a sample kit that includes four saliva collection tubes and a sample log. On the day before the next study visit, participants place a cotton swab underneath their tongue for 2 minutes to obtain a saliva sample and will repeat this four times throughout the day. Each time a sample is taken, the time is recorded by the participant on the sample log, along with additional questions about time since last cigarette, stress events, exercise level, and alcohol consumed on the collection day. Participants are asked to not eat or drink (especially caffeine or acidic drinks) or brush their teeth 30 minutes before taking a sample. Participants return the saliva kit at their visit the next day. During the visit, participants watch an instructional video [55] on how to collect and store the saliva samples. Participants who are chosen to complete the smoking topography protocol are provided with a handheld, portable smoking topography device called the Smoking Puff Analyzer-Mobile (SODIM SAS, Fleury-les-Aubrais, France). The device mechanically records measures of smoking behavior (e.g., number of puffs, puff flow, puff duration, inter-puff interval, and puff volume). The participant smokes through a mouthpiece connected by a tube to pressure transducers on the device. Participants are shown an instructional video [55] on how to operate and charge the device. For the next 2 days after the visit, they smoke their cigarettes with the device and return it at the next visit.

During Visit 2, participants are asked to submit their cigarette log from the prior week and to complete questionnaires. Biomeasures similar to Visit 1 are obtained, except for height, waist, and hip measurements (Fig. 2). Blood (~10 ml) and urine samples are collected for analysis at this visit and future visits (except Visit 11). All participants are given a 2- week supply of SPECTRUM research cigarettes containing a normal amount of nicotine (~11.6 mg) matching the flavor (regular or menthol) of their usual brand of cigarettes. The supply of research cigarettes given to the participant at each visit is equal to their reported CPD*number of days until the next visit (for Visit 2, this is 14 days), which is then multiplied by 150% to ensure an adequate supply is distributed until their next visit. Participants are asked to refrain from using other smoked or nicotine-containing products for the remainder of the study and to return all opened, unopened, and empty cigarette packs to the study center at each visit. Participants randomized to provide saliva return their samples to the study center, where they are given another sample kit to complete the day before the next study visit. Participants randomized to smoking topography return the device, and their smoking files are downloaded. The device is cleaned and redistributed for use on the next 2 days after the study visit.

#### Baseline Phase II: 2 weeks

During this phase, participants complete a phone call interview (lasting about 15 minutes) that consists of questionnaires on their cigarette log (including any use of other smoked or nicotine-containing products), cigarette liking, smoking urges, and withdrawal symptoms, as outlined in Fig. 2. Visit 3 is similar to Visit 2 and includes the same biological measures, completion of questionnaires, and collection of previous cigarette logs and cigarette packs (Fig. 2). At Visit 3, if participants agree to continue in the study and did not meet any of the early withdrawal criteria, they are randomized to either (1) smoke the same UNC research cigarettes (~11.6 mg) for 18 weeks or (2) switch to progressively RNC research cigarettes over 18 weeks (Table 2). Participants receive a 3-week supply of research cigarettes to last until their next visit along with cigarette logs to fill out showing daily cigarette consumption. Again, randomized participants return saliva samples and the smoking topography device, and a new sample kit and a clean device are given.

#### Randomization Phase: 18 weeks

During the Randomization Phase, participants return to the study center every 3 weeks for a total of six visits (Visits 4–9) to complete biomeasures and questionnaires as outlined in Fig. 2. Prior visit cigarette packs are collected. New research cigarettes equal to a 3-week supply corresponding to their treatment group are given at each visit. As with all study contacts, previous cigarette logs and information on use of other smoked or nicotine-containing products are collected. Any changes in health or medications are documented at each visit. Participants taking saliva samples and using the smoking topography device continue with the protocol until Visit 8. Participants are contacted for phone call interviews one week after Visits 4–8 to complete questionnaires as outlined in Fig. 2.

At Visit 9, the last visit of the Randomization Phase, participants are given the option as to how they would like to proceed with the next phase of the study. The three options are:

1. Return to their usual brand of cigarettes for 12 weeks (at their own cost)
2. Continue to receive research cigarettes for 12 weeks (provided at no cost)
3. Quit smoking with brief counseling from the study team and the option to use oral nicotine replacement therapy (NRT [2 mg gum or lozenges]) for 11 weeks (provided at no cost)

All participants receive a copy of the US Surgeon General Report, How Tobacco Smoke Causes Disease: What It Means to You [56] and local resources to help smokers quit. If participants choose to return to their usual brand of cigarettes, they are no longer given research cigarettes and are removed from the CMS, given additional cigarette logs, and are instructed to bring all logs back to the next study visit. If participants choose to continue on research cigarettes, they are given a 4-week supply of research cigarettes equivalent in nicotine content to the cigarettes they were given at the last visit, additional cigarette logs, and instructed to bring all logs and research cigarette packs back to the next study visit. If participants choose to quit smoking, they are given up to a 6-day supply of research cigarettes equivalent in nicotine content to the cigarettes they were given at the last visit and additional cigarette logs. These cigarettes are intended to last until their target quit date. Participants who choose to quit set a quit date approximately 1 week later and return to the study center after an approximately 24-hour period of abstinence so that the severity of their withdrawal can be assessed prior to dosing NRT. Additional information for the quit option is outlined below.

#### Treatment Choice Phase: 12 weeks

Regardless of the participant’s choice, all participants attend Visits 10 and 11 during the Treatment Choice Phase. During these visits, participants complete questionnaires and biomeasures as outlined in Fig. 2. Any changes in health or medications are documented. At Visit 10, participants who return to their usual brand continue on their own brand. Participants continuing to receive research cigarettes are given an 8-week supply of research cigarettes equivalent in nicotine content to the cigarettes they were given at the last visit. Participants still smoking return previous cigarette logs, and new logs are given. Participants making a quit attempt are offered a refill of NRT (three boxes of gum or lozenges) if needed and counseling on their quit attempt. At the final visit (Visit 11), all participants continuing to smoke return their cigarette logs and participants on research cigarettes return all packs. No more research cigarettes or NRT is given.

### Additional contacts for the quit option

Participants who choose to quit receive additional study contacts during the Treatment Choice Phase to facilitate a cessation intervention based on best practices developed for tobacco use. This includes cost-free behavioral interventions and FDA-approved medications for smoking cessation. Participants must be willing to set a quit date and are offered a flexible smoking cessation treatment. They have the option to receive up to 11 weeks of FDA-recommended doses of oral NRT (2 mg gum or lozenges) at no cost and cognitive behavioral-based smoking cessation counseling provided by study staff (in person or over the phone). In addition to the two regular study visits (Visit 10 [week 26] and Visit 11 [week 34]), there are two additional in-person counseling sessions (weeks 23 and 30), four phone counseling sessions (weeks 23, 24, 28, and 32), and five participant self-guided sessions. Participants receive 20 minutes (or less) at each session of standard individual cognitive behavioral therapy based on the Freedom from Smoking curriculum [57] from the American Lung Association. Standardized supplemental materials on cessation are provided.

### Compensation

Participants receive a $20 gift card at each study visit to cover parking, meals, or travel to the 11 study visits ($220). Participants receive $20 for the first and last study visits. Participants receive $60 for completing each of the remaining 9 study visits ($580). Participants receive $10 for each of the seven phone surveys they complete ($70). If participants complete all study visits and return used and unused cigarette packs and study equipment (e.g., the smoking topography device) they receive a $130 study compliance payment at the last visit. The total compensation is $1000.

Participants who used the smoking topography device throughout the study receive an additional one-time check payment of $60 for completing this extra protocol at the last study visit. Participants who provided saliva samples throughout the study receive an additional one-time check payment of $35 for completing this protocol at the last study visit. If the participant does not complete the protocol, they do not receive payment.

### Sample size

It is estimated that we will recruit 400 participants (200 white and 200 black smokers), allowing for a 30% post randomization withdraw rate, to allow for 280 participants completing the entire protocol. Sample size calculations are based on a secondary aim, total plasma cotinine concentration (measured as nanograms per milliliter). A total sample size of 280 participants (70 per group* 2 treatment groups* 2 races) will enable us to detect a mean cotinine difference of 68 ng/ml as observed in the Benowitz et al. [36] trial with at least 90% power.

### Data management and monitoring

All study data are collected and managed across sites using Research Electronic Data Capture (REDCap) [58] tools hosted at the Penn State Milton S. Hershey Medical Center and College of Medicine. REDCap is a secure, web-based application designed to support data capture for research studies. Access to the REDCap database requires double authentication (two unique usernames and passwords), and a user matrix is used to ensure that only appropriate data are accessed based on the individual’s role in the project. Any paper records generated during the trial will be stored in locked file cabinets in areas with limited access. Trial data will undergo internal auditing by an independent data management team at Penn State University. The Data Monitors will review source documentation to determine whether the data reported in the web-based system are complete and accurate and verify that standard operating procedures and policies are followed during study implementation.

Adverse event reporting and review is ongoing throughout the trial. The Safety Monitor (Rebecca Bascom) oversees the safety of the participants in the trial and assesses the causality of all adverse events. Adverse events will be reported to the applicable institutional review boards (IRBs) in accordance with IRB policies and procedures. All serious adverse events are reported within 5 days to the FDA’s Center for Tobacco Products Safety Reporting Portal. The Monitor receives summary data safety monitoring reports on recruitment, retention, adverse events, cigarettes per day, and CO, and produces bi-annual reports and recommendations on the continuation of the research to the principal investigators.

### Statistical analysis

Basic baseline statistics including means (standard deviations) and frequency distributions (percentages) will be reported for demographic characteristics, smoking characteristics, nicotine dependence, and nicotine and toxicant exposure. Characteristics will be reported by the two groups under investigation to identify treatment group imbalances. Numerical baseline characteristics will be compared between the two groups using two-sample *t* tests or non-parametric Wilcoxon rank sum tests when appropriate. Suitably transformed variables will be used when necessary. Categorical variables will be compared using chi-square tests or Fisher’s exact tests. The analysis of all endpoints will adhere to the intention-to-treat principle, where all randomized participants will be included in the analysis regardless of compliance or study completion.

The frequency of smokers who drop out and the degree of non-compliance will be summarized for each treatment group. The levels of within-subject cotinine normalized for the number of cigarettes smoked will be used to measure the degree of compliance using the RNC cigarettes ([plasma cotinine/CPD (end of randomization)]/[plasma cotinine/CPD (baseline)]) as first described by Benowitz et al. (2015) [40]. Self-report measures of use of other nicotine-containing products including usual brand cigarettes will also be assessed. The two-sample *t* test or nonparametric Wilcoxon rank sum test will be used to compare the nicotine metabolite measurements between groups with and without drop-out/relapse. Fisher’s exact test will be used to compare the drop-out/relapse rate between the two groups. A multi-variable logistic regression model will be built to examine the significance of the preceding factors on the drop-out/relapse rate. The magnitude of the effectiveness of each factor will be quantified by the estimated odds ratio with its 95% confidence interval. In addition, the sensitivity, specificity, and positive and negative predictive values in using these predictors to classify the outcome (drop-out versus non drop-out) will be estimated. The interaction between the predictors will also be explored in the analysis.

Each of the secondary outcome variables of interest will be analyzed within and across time periods. Results will be summarized by tables and figures (such as boxplots). Profile plots will be generated to show the trajectory of variables across different time periods. The major analytical tool for addressing the specific aims of this study is linear mixed models with repeated measures. For each biomarker and other numerical outcome measures of interest, a linear mixed repeated measures model will be fit to evaluate the main effects of time, group, and time-by-group interaction, treating baseline scores for outcomes as covariates. Known confounders will be included in the linear models, and other covariates will be included if their individual bivariate associations with both the group variable and outcome are significant at a 10% level. A subgroup analysis will be performed by race. We will also test for a number of potential effect modifiers by pooling the trial data of white smokers and black smokers.

To determine modification effects, such as menthol, the menthol-by-group interaction in the multi-variable linear mixed model will be examined. The three-way interaction of menthol-by-group-by-time will be explored but will be removed from the model if not significant. If the menthol-by-group interaction is found significant, then mean-profile plots will be used to show the interaction in detail.

Stress measures will be examined by both salivary biomarkers and psychological questionnaires. The mean cortisol values (average of four measurements) over different time points will be converted to area under the curve (AUC), which is an aggregate index based on repeated measures over time of day. Two different calculations will be made; one is AUCg (ground), which measures total area under the curve, and the other is AUCi (increase), which measures change beyond baseline over time. We will use *t* tests to compare salivary cortisol and alpha amylase measures between the two study groups at each time point.

Analyses will be conducted by the Penn State TCORS Biostatistics Core using statistical software SAS version 9.4 or higher (SAS Institute, Cary, NC, USA) and the R programming language version 3.3.2 or higher (R Foundation). The smoking topography data will be preprocessed (before statistical analysis) using the Python programming language version 2.7 (Python Software Foundation). All tests will be two-sided, and the statistical significance level to be used is 0.05. Results pertaining to study outcomes will be reported on ClinicalTrials.gov.

## Discussion

The purpose of the Penn State TCORS is to provide evidence-based policy research on tobacco regulatory actions. There are various levels of nicotine content in reduced nicotine cigarettes, and empirical data are needed to determine the optimal strategies for reducing cigarette exposure, including the dosing, time and duration of doses, individual variation, and potential harm from nicotine reduction. This study focuses on progressively switching smokers from a high nicotine commercial cigarette to a very low nicotine content cigarette in a tapered fashion over time. Progressively reducing nicotine might facilitate the ability to switch to low nicotine cigarettes while minimizing any potential harm. One of the major goals of the Penn State TCORS is to evaluate RNC cigarettes in vulnerable populations who are affected the hardest by the detrimental health effects of smoking and the degree of nicotine dependence. Tobacco use and dependence are highly associated with low SES, homelessness, imprisonment, and mental health disorders [7, 59–61]. Consequently, research on a potential national policy that includes these populations should establish feasibility and safety. A reduced nicotine strategy may positively impact low SES populations who disproportionately consume tobacco and experience its deleterious health effects. Clinical trial data of the benefits and harms of RNC cigarettes are used by the FDA in the evaluation process of lowering nicotine in cigarettes.

### Trial status

The trial started recruiting participants in August 2015 and is currently enrolling participants. Trial findings are likely to be available in early 2019.

## Additional file

Additional file 1: SPIRIT 2013 checklist: recommended items to address in a clinical trial protocol and related documents. (DOC 121 kb)

## Abbreviations

AUC: Area under the curve
CMS: Cigarette Management System
CO: Carbon monoxide
FDA: Food and Drug Administration
IRB: Institutional review board
ITP: Investigational Tobacco Product
NRT: Nicotine replacement therapy
REDCap: Research Electronic Data Capture
RNC: Reduced nicotine content
SES: Socioeconomic status
TCA: Family Smoking Prevention and Tobacco Control Act
TCORS: Tobacco Center of Regulatory Science
UNC: Usual nicotine content
