# Correction to Reduced nicotine content cigarettes in smokers of low socioeconomic status: study protocol for a randomized control trial

**DOI:** 10.1186/s13063-017-2356-y

**Published:** 2017-12-15

**Authors:** Nicolle M. Krebs, Sophia I. Allen, Susan Veldheer, Diane J. Martinez, Kimberly Horn, Craig Livelsberger, Jennifer Modesto, Robin Kuprewicz, Ashley Wilhelm, Shari Hrabovsky, Abid Kazi, Alyse Fazzi, Jason Liao, Junjia Zhu, Emily Wasserman, Samantha M. Reilly, Lisa Reinhart, Neil Trushin, Robinn E. Moyer, Rebecca Bascom, Jonathan Foulds, John P. Richie, Joshua E. Muscat

**Affiliations:** 1 Department of Public Health Sciences, Penn State Tobacco Center of Regulatory Science, Pennsylvania State University, MC CH69, 500 University Drive, P.O. Box 850, Hershey, PA 17033 USA; 2 Investigational Drug Service, Department of Pharmacy, Pennsylvania State University, 500 University Drive, P.O. Box 850, Hershey, PA 17033 USA; 3 The Milken School of Public Health, George Washington University, 950 New Hampshire Ave, NW, Washington, D.C, 20052 USA; 4 Department of Medicine, Pennsylvania State University, 500 University Drive, P.O. Box 850, Hershey, PA 17033 USA

## Correction

The title of the original publication [1] had an error; furthermore there were errors in Fig. 2. The corrected version of the title and of Fig. 2 (Additional file 1 here) can be found below in this Erratum.

Incorrect title:

Reduced nicotine content cigarettes in smokers of low socioeconomic status: study protocol for a randomized control trial

Correct title:

Reduced nicotine content cigarettes in smokers of low socioeconomic status: study protocol for a randomized controlled trial

Error in Fig. 2:

The study week and study day numbers at the top of the figure should match up with the numbers at the bottom of the figure.

## Additional file

Additional file 1: SPIRIT figure. (DOCX 48 kb)
