# Supplementary material for: Correction to Reduced nicotine content cigarettes in smokers of low socioeconomic status: study protocol for a randomized control trial
Source: Trials. 2017 Dec 15;18:598. doi: 10.1186/s13063-017-2356-y (PMC5732394; doi:10.1186/s13063-017-2356-y)
Supplement: Additional file 1: — SPIRIT figure. (DOCX 48 kb) [file 13063_2017_2356_MOESM1_ESM.docx]

|  | **Baseline Phase I** | | **Baseline Phase II** | | **Randomization Phase** | | | | | | | | | | | | **Treatment Choice Phase** | |
| --- | --- | --- | --- | --- | --- | --- | --- | --- | --- | --- | --- | --- | --- | --- | --- | --- | --- | --- |
| **Study Week Number** | **0** | **1** | **2** | **3** | **4** | **6** | **7** | **9** | **10** | **12** | **13** | **15** | **16** | **18** | **19** | **21** | **25** | **33** |
| **Study Day** | 1 | 7 | 14 | 21 | 28 | 42 | 49 | 63 | 70 | 84 | 91 | 105 | 112 | 126 | 133 | 147 | 175 | 231 |
| **Study Visit Number** | 1 | 2 | Ph | 3 | Ph | 4 | Ph | 5 | Ph | 6 | Ph | 7 | Ph | 8 | Ph | 9 | 10 | 11 |
| **Measures/Questionnaires** |  |  |  |  |  |  |  |  |  |  |  |  |  |  |  |  |  |  |
| Daily cigarette and other nicotine product use log |  | X | X | X | X | X | X | X | X | X | X | X | X | X | X | X | X | X |
| Concomitant medications | X | X |  | X |  | X |  | X |  | X |  | X |  | X |  | X | X | X |
| Adverse events [65] |  | X |  | X |  | X |  | X |  | X |  | X |  | X |  | X | X | X |
| Demographics [66] | X |  |  |  |  |  |  |  |  |  |  |  |  |  |  |  |  |  |
| Tobacco use history | X |  |  |  |  |  |  |  |  |  |  |  |  |  |  |  |  |  |
| NIDA drug screening tool [67] | X |  |  |  |  |  |  |  |  | X |  |  |  |  |  | X |  |  |
| Environmental smoke questionnaire [68] |  | X |  | X |  | X |  | X |  | X |  | X |  | X |  | X | X | X |
| Perceived health risk |  | X |  | X |  |  |  |  |  | X |  |  |  |  |  | X |  |  |
| Cigarette liking scales [33, 69, 70] | X | X | X | X | X | X | X | X | X | X | X | X | X | X | X | X | X | X |
| Nicotine dependence questionnaires [71-75] | X | X |  | X |  | X |  | X |  | X |  | X |  | X |  | X | X | X |
| Minnesota nicotine withdrawal scale [76] | X | X | X | X | X | X | X | X | X | X | X | X | X | X | X | X | X | X |
| Questionnaire on smoking urges [77, 78] | X | X | X | X | X | X | X | X | X | X | X | X | X | X | X | X | X | X |
| Audit-C alcohol use [79, 80] |  | X |  |  |  |  |  | X |  |  |  | X |  |  |  | X |  |  |
| Cigarette brand details | X | X |  |  |  |  |  |  |  |  |  |  |  |  |  |  |  |  |
| Center for Epidemiologic Studies- Depression [81] | X | X |  | X |  | X |  | X |  | X |  | X |  | X |  | X | X | X |
| Kessler 6 scale [82, 83] | X | X |  | X |  | X |  | X |  | X |  | X |  | X |  | X | X | X |
| Perceived stress scale [84] | X | X |  | X |  | X |  | X |  | X |  | X |  | X |  | X | X | X |
| Clinical COPD questionnaire [85] |  | X |  | X |  | X |  | X |  | X |  | X |  | X |  | X | X | X |
| Menthol questionnaire [86] |  | X |  | X |  | X |  | X |  | X |  | X |  | X |  | X | X |  |
| Packaging questionnaire | X |  |  | X |  |  |  |  |  |  |  |  |  |  |  | X |  |  |
| Wisconsin predicting patients’ relapse questionnaire (partial)[87] | X |  |  |  |  |  |  |  |  |  |  |  |  |  |  | X |  |  |
| Smoking cessation questionnaire (if chosen by participant) [88] |  |  |  |  |  |  |  |  |  |  |  |  |  |  |  |  | X | X |
| **Biomeasures/Procedures** |  |  |  |  |  |  |  |  |  |  |  |  |  |  |  |  |  |  |
| Weight [89] | X | X |  | X |  | X |  | X |  | X |  | X |  | X |  | X | X | X |
| Height [89] | X |  |  |  |  |  |  |  |  |  |  |  |  |  |  |  |  |  |
| Waist:hip ratio [89] | X |  |  |  |  |  |  |  |  |  |  |  |  |  |  | X |  |  |
| Exhaled carbon monoxide [90] | X | X |  | X |  | X |  | X |  | X |  | X |  | X |  | X | X | X |
| Blood pressure/pulse [91] | X | X |  | X |  | X |  | X |  | X |  | X |  | X |  | X | X | X |
| Pregnancy test | X |  |  | X |  |  |  | X |  |  |  | X |  |  |  | X | X | X |
| Urine specimen collection |  | X |  | X |  | X |  | X |  | X |  | X |  | X |  | X | X |  |
| Blood specimen collection |  | X |  | X |  | X |  | X |  | X |  | X |  | X |  | X | X |  |
| DNA extraction |  | X |  |  |  |  |  |  |  |  |  |  |  |  |  |  |  |  |
| Saliva sample collection (100 randomly selected participants) |  | X |  | X |  | X |  | X |  | X |  | X |  | X |  |  |  |  |
| Smoking topography collection (100 randomly selected participants) |  | X |  | X |  | X |  | X |  | X |  | X |  | X |  |  |  |  |
| Compensation | $20 | $60 | $10 | $60 | $10 | $60 | $10 | $60 | $10 | $60 | $10 | $60 | $10 | $60 | $10 | $60 | $60 | $20 |
| **Study Week Number** | **0** | **1** | **2** | **3** | **4** | **6** | **7** | **9** | **10** | **12** | **13** | **15** | **16** | **18** | **19** | **21** | **25** | **33** |
| **Study Day** | 1 | 7 | 14 | 21 | 28 | 42 | 49 | 63 | 70 | 84 | 91 | 105 | 112 | 126 | 133 | 147 | 175 | 231 |
| **Study Visit Number** | 1 | 2 | Ph | 3 | Ph | 4 | Ph | 5 | Ph | 6 | Ph | 7 | Ph | 8 | Ph | 9 | 10 | 11 |
| Abbreviations: COPD= chronic obstructive pulmonary disease, DNA= deoxyribonucleic acid, NIDA= National Institute on Drug Abuse, Ph= Phone call interview | | | | | | | | | | | | | | | | | | |
